# Cervical Spine Involvement in Mild Traumatic Brain Injury: A Review

**DOI:** 10.1155/2016/1590161

**Published:** 2016-07-26

**Authors:** Michael Morin, Pierre Langevin, Philippe Fait

**Affiliations:** ^1^Department of Human Kinetics, Université du Québec à Trois-Rivières (UQTR), Trois-Rivières, QC, Canada G9A 5H7; ^2^Research Group on Neuromusculoskeletal Dysfunctions (GRAN), UQTR, Trois-Rivières, QC, Canada G9A 5H7; ^3^Cortex Médecine et Réadaptation Concussion Clinic, Quebec City, QC, Canada G1W 0C5; ^4^Department of Rehabilitation, Faculty of Medicine, Laval University, Quebec City, QC, Canada G1V 0A6; ^5^Research Center in Neuropsychology and Cognition (CERNEC), Montreal, QC, Canada H3C 3J7

## Abstract

*Background*. There is a lack of scientific evidence in the literature on the involvement of the cervical spine in mTBI; however, its involvement is clinically accepted.* Objective*. This paper reviews evidence for the involvement of the cervical spine in mTBI symptoms, the mechanisms of injury, and the efficacy of therapy for cervical spine with concussion-related symptoms.* Methods*. A keyword search was conducted on PubMed, ICL, SportDiscus, PEDro, CINAHL, and Cochrane Library databases for articles published since 1990. The reference lists of articles meeting the criteria (original data articles, literature reviews, and clinical guidelines) were also searched in the same databases.* Results*. 4,854 records were screened and 43 articles were retained. Those articles were used to describe different subjects such as mTBI's signs and symptoms, mechanisms of injury, and treatments of the cervical spine.* Conclusions*. The hypothesis of cervical spine involvement in post-mTBI symptoms and in PCS (postconcussion syndrome) is supported by increasing evidence and is widely accepted clinically. For the management and treatment of mTBIs, few articles were available in the literature, and relevant studies showed interesting results about manual therapy and exercises as efficient tools for health care practitioners.

## 1. Introduction

Mild traumatic brain injury (mTBI) is commonly known as concussion [1]. In a recent study, Statistics Canada estimated mTBI annual incidence to be 600 per 100,000 people [1]. The pediatric TBI population is the patients' subgroup who consulted the most often in the emergency room [1]. An estimated 1.6 to 3.8 million sport and recreation-related brain injuries occur in the United States annually, and up to 75% of them are classified as mild [2]. About 70 to 90 percent of all TBI cases are thought to be of mild severity, and the related symptoms usually resolve within 7 to 10 days [3, 4]. However, as many as 50% of concussions may go unreported [5]. In an epidemiologic study, Tator et al. (2007) reported that the highest incidence for TBI is in the age group under 18 years, with almost 45%. Additionally, approximately one-quarter of all patients with TBI are aged between 19 and 29 years [1]. The Centers for Disease Control and Prevention (CDC) describes mTBI as a silent epidemic [2].

Concussion in sports was defined in 2012 at the international consensus on concussion in sport held in Zurich as

*a brain injury and is defined as a complex pathophysiological process affecting the brain, induced by biomechanical forces. Several common features that incorporate clinical, pathologic and biomechanical injury constructs may be utilized in defining the nature of a concussive head injury. This is caused by a direct blow to the head, face, neck or elsewhere on the body with an “impulsive” force transmitted to the head. [4]*

The number of reported concussions has increased in recent years for multiple reasons. Recent studies identified an increased awareness of the potential complications following concussion and repeated head trauma in the population [6] and higher involvement of health professionals in sports and in concussions recognition and follow-up [7]. Even though it is believed that most concussions usually resolve in between 7 and 10 days [3, 4], the symptoms may persist longer for up to 33 percent of cases [8]. Symptoms persisting for a few weeks to more than six months are defined in the literature as postconcussion syndrome (PCS) [8].

PCS is a complex medical subject that few articles and studies in the scientific literature explore concretely [4]. Considering that mTBI is a multifaceted injury, many signs and symptoms complicate its diagnosis [3]. Multiple impairments linked to this condition such as cognitive, vestibular, cervical, physical, and psychological dysfunctions [3, 4]. Therefore, with a multitude of clinical theories in the literature, it is difficult to determine clinical guidelines for PCS [3]. Current consensus identifies that a multidisciplinary approach is essential to the progress of the patient suffering from PCS [4].

One of the major challenges in the medical management of concussion is that there is no “gold standard” for assessing and diagnosing the injury [9]. It has been shown by Schneider et al. (2014) that a combination of cervical and vestibular physiotherapy decreased time to medical clearance for the return to sport in a cohort of 31 patients (12–30 years old) with persistent symptoms of dizziness, neck pain, and/or headaches following a sport-related concussion [10]. However, little research has been published on this very specific topic.

## 2. Problem

The problem targeted by this review of literature is the lack of data regarding the association between mTBI and cervicogenic impairment. The majority of scientific publications focus on diagnosing mTBI, and there is little evidence on the possible involvement of the cervical region [4]. Cervical spine examination after a cranial trauma is essential, but the association between symptoms and mTBIs is poorly described in the literature. A literature review will improve the medical knowledge of all medical professionals and help the diagnosis and treatment of mTBI.

## 3. Goals

The aim of this review was to target scientific articles describing mTBI and those involving cervicogenic headache (CGH) cases in order to look at the connections and similarities between these two types of injuries. The specific goals of this paper are (1) to determine the common signs and symptoms of mTBI (including PCS) and cervical dysfunctions, (2) to describe the mechanism of cranial trauma injury and link it with the impact on the cervical spine, and (3) to give an update on the various types of effective treatments for these conditions.

## 4. Methods

The following electronic resources were searched from January 1, 1990, to May 19, 2015:* PubMed, Index to Chiropractic Literature (ICL), SportDiscus, Physiotherapy Evidence Database (PEDro), Cumulative Index to Nursing and Allied Health Literature (CINAHL)*, and* Cochrane Library databases*. The following keywords were used in different combinations:* concussion*,* neck*,* TBI*,* mTBI*,* cervical*,* physiotherapy*,* physical therapy*,* athletic training*,* treatment*,* chiropractic*,* manipulation*,* manual therapy,* and* guideline*. For the complete list of combinations, see Table 1. Three data collections were carried out at different dates: February 16, 2014, May 15, 2014, and May 19, 2015. Reference lists of articles meeting the selection criteria were also collected. All abstracts in English and French dealing with concussion involving the neck were selected for a full reading of the article. A total of 4,854 abstracts were found on search engines. Following this search, the articles were selected according to three main streams:

1. mTBI's and PCS symptoms related to the cervical spine.
2. Mechanism of injury.
3. Therapies of the cervical spine with symptoms related to mTBI.

**Figure 1:**
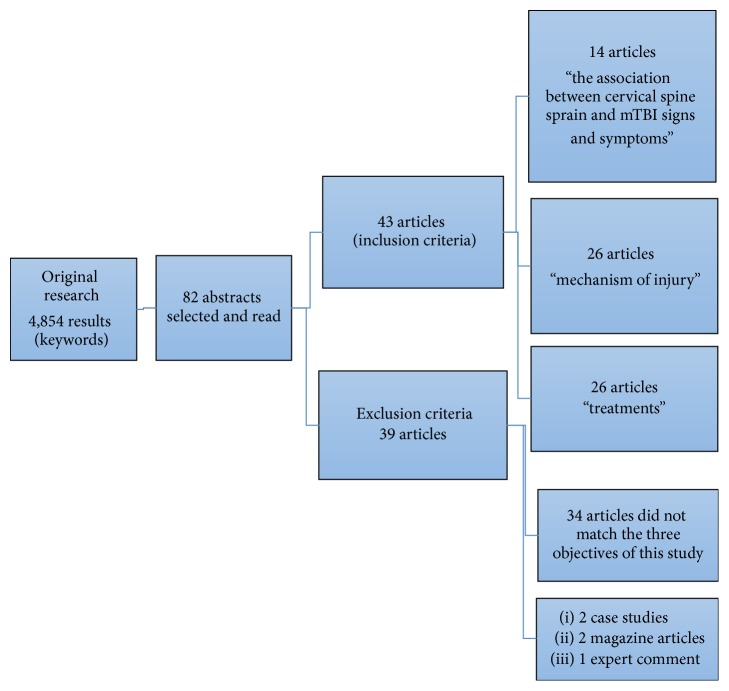
Research method for this review.

Inclusion criteria, based on the precited keywords, were original data articles, literature reviews, and clinical recommendations, available in English and/or French, based on the objectives of this research. Exclusion criteria were foreign language papers other than English or French, case studies, magazine articles, and expert and editorial comments. The selection of articles is shown in Figure 1, and combinations are explained in Table 1.

## 5. Results

After a careful screening of 4,854 data entries, 82 abstracts met the inclusion criteria of our research and were reviewed. All these articles were read and analyzed. Thirty-eight articles were excluded because the main topic did not match the three topics of this study (*n* = 34) or were case studies (*n* = 2), magazine articles (*n* = 2), and expert commentary (*n* = 1). Finally, 43 papers were included in this literature review. Each selected article is described in Table 2 (see Appendix). These articles were analyzed and assigned to the different categories of topics discussed: (1) the association between cervical spine sprain and mTBI signs and symptoms, (2) mechanism of injury, and (3) treatments. Results are represented in Figure 1 and Table 1.

## 6. Discussion

### 6.1. The Association between Cervical Spine Sprain and mTBI Signs and Symptoms

The 2012 Zurich consensus on concussion in sports led by McCrory et al. (2013) defined a list of 22 common symptoms of mTBI divided into 4 main categories, as described in Table 3 [4, 6, 11–13]. The majority of mTBIs (80–90%) are resolved in 7 to 10 days [4, 14, 15]. However, in the remaining 10 to 20%, symptoms may persist for more than 10–14 days, and even for several months after trauma [4, 16]. This clinical picture is diagnosed as PCS and the etiology for PCS is not well defined in the literature [11, 14, 17].

As mentioned previously, the most common symptom in subjects after mTBI is posttraumatic headache (PTH) [18, 19]. The incidence of PTH varies between 5 and 90% [16, 20]. Their prevalence in children with mTBI is from 73 to 93% [19]. The diagnosis of PTH can be difficult to address in subjects with a history of preexisting headaches. However, PTH syndrome diagnosis is considered when the intensity or frequency of headaches increases after trauma [19]. PTH is defined as a headache that occurs within 1 week after regaining consciousness or within 1 week following head trauma [17]. The majority of PTH resolve within 6 to 12 months, and it is caused by cervical muscle tension and posture impairment [17]. Because headache is one of the major causes of morbidity in mTBI subjects, health care professionals should manage this symptom with a high level of priority [17].

Biologically, concussions usually resolve 7 to 10 days after trauma in adults [4, 21]. PCS related symptoms are nonspecific. Professionals must consider other pathologies as alternative explanations to persistent symptoms [4, 17]. Meanwhile, a recent study suggests that even at 1 month or more after concussion, the cerebral blood flow is decreased in 36% of 11- to 15-year-old subjects who suffered mTBI compared to a nonconcussed control group [22]. Further studies are needed to clarify this phenomenon.

Rather than a structural problem, PCS would be related to a brain dysfunction problem [15]. This would explain the negative results on most of the medical imaging prescribed in emergencies [15]. In addition, a prospective Norwegian study of 348 participants identified through a questionnaire that headaches persisting for more than 3 months after trauma and diagnosed as PCS are often related to a musculoskeletal pathology. In other words, the head or brain injury does not cause the persistent symptoms [23]. These results show the importance of potential cervical impairment in patients with mTBI [4, 14, 16, 17].

### 6.2. Mechanism of Injury

Recent studies correlated mTBI with whiplash occurrence [12, 16, 24, 25]. Furthermore, Schneider et al. (2013) demonstrated in a prospective study with 3,832 male ice hockey players (11–14 years old) that the presence of headache and neck pain in a preseason evaluation increases the risk of concussions during the season [26]. Consequently, a neck examination should be part of the postconcussion follow-up in addition to the neurological screening examination [6].

Whiplash is defined as a mechanism of acceleration-deceleration transferred to the cervical spine [6, 20, 27, 28] (see Table 4). With its large range of motion, the upper cervical spine is the most mobile part of the spine. During a whiplash, cervical structures are stressed at their end ranges of motion which can lead to neck injuries [29]. The impact generates stresses and injuries in the bones and soft tissues of the cervical spine, causing clinical manifestations [24]. Among those, whiplash symptoms include neck pain, cervicogenic headaches, chest pain, memory and concentration disturbances, muscle tension, sleep disturbances, dizziness, fatigue, cervical range of motion restrictions, irritability, tinnitus, and visual disturbances [12, 17, 20, 24, 25]. A preliminary study has demonstrated that low velocity impacts between 4 and 12 km/h can provoke neck and head injuries causing dysfunctions and pain [12]. These symptoms are often similar to those listed for mTBI, which leads to confusion for the medical community [12, 14, 16, 25].

The pathophysiology related to the patient's symptoms originates from one or several structures of the cervical spine. After a trauma involving the cervical region, the involvement of muscles, ligaments, arteries, nerves, the esophagus, the temporomandibular joint, intervertebral discs, zygapophysial joints, vertebrae, and the atlantooccipital joint creates a complex challenge for clinicians [16, 17, 20].

One of the hypotheses raised in the literature for the origin of neck pain is the involvement of the cervical zygapophysial joints [30]. Zygapophysial joints have been shown to be the source of neck pain, headaches, visual disturbances, tinnitus, and dizziness in patients who have sustained whiplash [14, 24, 25]. In this following order, the C1-C2, C2-C3, C0-C1, and C3-C4 levels are the most often described in association with cervical symptoms following mTBI [16, 24, 26, 31]. More specifically, cervical zygapophysial joint pain is expressed by a hypersensitivity and hyperexcitability of the spinal cord reflexes, causing an increase of nociceptive processes in the central nervous system [32]. The cross-sectional study of Smith et al. (2013) recruited 58 adults (18–52 years old) with chronic whiplash disorder and provided a facet joint block to all participants [32]. They analyzed characteristics of responders and nonresponders to facet joint block and concluded that patients with chronic Whiplash-Associated Disorder (WAD) show similar sensory disturbances, motor dysfunction, and psychological distress [32]. Another study published by Treleaven et al. (1994) supports the implication of the cervical region by a precise physical examination in differential diagnosis of 12 patients suffering of persistent postconcussion headache [31].

Following a whiplash, the cervical spine is often the source of a patient's pain [4, 10]. The neck received the transmitted force of the impact and the same acceleration-deceleration mechanism that produces mTBI [28]. During the whiplash, the cause of cervicogenic and brain induced symptoms could be caused by either the mTBI or the cervical spine involvement or both at the same time [10]. There are similarities between the symptoms of neck disorders and the symptoms of mTBI [19]. The most common posttraumatic symptom is headache [4]. Cervicogenic headache and posttraumatic headache are well-known conditions [4, 13, 19, 33]. In fact, those upper cervical impairments, if not diagnosed and treated, can lead to chronicity of postconcussion headaches [10].

Cervicogenic headaches are common after a whiplash injury [16, 34]. Different studies showed that 3 to 4.6% of patients will develop chronic daily headaches after whiplash and 2% will be permanently disabled [34]. Upper cervical spine pain can arise from various anatomical structures such as muscles, joints, ligaments, and nerves [34]. Tensions in cervical muscles (trigger points) are the most common diagnosed type of headache [16, 23]. Becker (2010) explained that headaches related to cervical spine disorders (CGH) remain one of the most controversial areas of headache medicine [34]. Dysfunctions of the craniocervical zygapophysial (C0 to C4) joints can also cause headaches [16, 30]. In patients with headaches following a whiplash injury, dysfunctions of the C2-C3 zygapophysial joint are highly prevalent, particularly if there is tenderness over the C2-C3 facet joint [34]. Cervicogenic headaches may be unilateral or bilateral with the dominance depending on one or more of the structures that are affected [16]. Pain location usually begins in the occipital region of the neck [34]. After a whiplash injury, zygapophysial joints are clinically identified as the single most common source of pain in at least 50% of neck pain. Furthermore, facet joints appear to be the most common source of pain in the neck, with or without headache [34]. King et al. (2007) showed in their retrospective study, which included 173 patients, that manual examination had a high degree of sensitivity during zygapophysial joint pain evaluation [35]. Tension in the cervical muscles has the potential effect of reducing neck movement and generating local pain [16, 18]. A bad posture or sleeping position as well as physical activity performed with a faulty motor strategy can lead to neck pain and pain irradiating to the head [19]. In 2013, the Cervicogenic Headache International Study Group (CHISG) developed the diagnostic criteria for CGH [33] (see the following).

*Summary of the Cervicogenic Headache (CGH) Diagnostic Criteria [33]*

 Unilaterality of pain, although it is recognized that bilateral cervicogenic headache may occur.
 Restriction in range of motion in the neck.
 Provocation of usual head pain by neck movement or sustained awkward neck positions.
 Provocation of usual head pain with external pressure over the upper cervical or occipital region on the symptomatic side.
 Ipsilateral neck, shoulder, or arm pain, usually of a vague nonradicular nature, occasionally radicular.

Posttraumatic headaches are a serious contributor to disability following cranial, cerebral, and cervical injury. Therefore, evaluating the cervical region after a head trauma is recommended [4]. This recommendation will highly contribute to limiting morbidity of mTBI and, furthermore, to help clinicians identify the International Headache Society (IHS) cervicogenic characteristics on mTBI patients. One of the improvements of SCAT3 and Child-SCAT3 compared to the SCAT2 is the introduction of the cervical assessment in posttrauma evaluation [4]. Knowing that the mechanism of mTBI is an external force transmitting energy to the head, it is possible that the neck, supporting the head, can also be injured during such an external force [11, 12, 16]. The Zurich consensus for concussion in sports (2012) recommended a multidisciplinary approach for patients who suffer PCS symptoms, such as headaches lasting longer than 6 weeks [4].

### 6.3. Treatments

The majority of articles in the literature currently focus on the diagnosis of mTBI, but few are dedicated to its management and treatments [7]. The 2012 Zurich consensus on concussion in sport suggested physical and cognitive rest until the end of the acute symptoms after trauma and a multidisciplinary approach involving experienced health care professionals when treating mTBI [4]. In their retrospective analysis, Moser et al. (2012) analyzed 49 high school and collegiate athletes (mean = 15.0 years old) and suggested that a period of cognitive and physical rest may be a useful mean of treating concussion-related symptoms [21]. This recovery time allows for a period of 7 to 10 days before the athlete returns to competition [2]. During this period, the symptoms should be evaluated daily and all activities that increase these symptoms should be stopped [4]. A Cochrane study mentioned that, based on present literature, no acutely initiated intervention has been clearly associated with a positive outcome for patients who sustain mTBI [36].

Return-to-play is allowed when athletes are symptom-free at rest, are able to do a full practice with contact without symptoms, no longer take any medications, and have returned to their baseline levels of cognitive functioning and postural stability [6].

The evaluation of the cervical region has been included as a new part of the SCAT3/Child-SCAT3, and a full clearance is essential before return-to-play [4]. According to some authors, post-mTBI subjects must have no pain in the neck, full mobility, and an adequate bilateral general strength to restart their sporting activities [12].

Treatments such as vertebral manual therapy, cervical tractions, manipulations, and exercises can relieve neck pain [16]. Brolinson (2014) has demonstrated that interventions of spinal manual therapy, physiotherapy, and neuromotor/sensorimotor training are more effective for mTBI recovery compared to a program of rest and exercises [7]. Another study demonstrated that the physical status of individuals with neck pain is improved with an exercise program combining manipulation, proprioceptive neuromuscular facilitation, acupressure on trigger points, and range of motion exercises, along with proprioceptive exercises compared to a neck pain control group of similar patients treated with information and advice [29]. Treatment of the cervical spine (sustained natural apophyseal glides) has been shown to be effective in 17 individuals with suspected cervicogenic dizziness compared to a control group (17 adults) [37]. Schneider et al. (2014) established that a significantly higher proportion of post-mTBI individuals (more than 3 weeks after trauma) were medically cleared to return to sport within 8 weeks of initiating treatment if they were treated in physiotherapy with cervical spine and vestibular rehabilitation compared to a control group [10]. Another study recruited 128 mTBI adults with either PCS or cervicogenic/vestibular symptoms [27], who completed the 22-symptom postconcussion symptoms scale questionnaire. Their results demonstrated that the questionnaire does not reliably discriminate between both types of patients. They concluded that clinicians should consider specific testing of exercise tolerance and perform a physical examination of the cervical spine and the vestibular/ocular systems to determine the etiology of postconcussion symptoms and to consider treating these accordingly [27]. Assessment and treatment of the cervical spine and vestibular system in the presence of persistent dizziness, neck pain, and/or headaches may facilitate functional and symptomatic improvements and shorten recovery in post-mTBI subjects [7, 10].

There is little evidence in clinical trials on the treatment of PTH. A study by Bonk et al. (2000) has shown that physiotherapy treatments decrease pain and increase cervical range of motion compared to a control group in a cervical collar for a period of 6 and 12 weeks after trauma [38]. The physiotherapy programs consisted of active and passive mobilizations, postural strengthening, the application of ice, and exercises that are efficient for PTH.

Regarding the cervical muscular system, several changes are observed following a trauma such as mTBI or whiplash. There is still nonempirical support that stronger neck muscles could reduce the risks of mTBI on the field [11, 39]. In fact, Mihalik et al. (2011) evaluated the effect of cervical strength on head impact in 37 hockey players (average 15 years old) and they concluded that the hypothesis of neck strength decreasing head acceleration was not supported [39]. Recent publications identify the importance of neck musculature in the prevention of concussions. Two hypotheses are presently under debate in the literature. The potentially modifiable risk factors for concussion are neck strength and impact anticipation [40]. Tierney et al. (2005) demonstrated that males have a better head-neck segment dynamic stabilization than females when angular acceleration is sustained by the head in a study including 20 males and 20 females [41]. Furthermore, a descriptive study demonstrated that greater neck strength and anticipatory cervical muscle activation (bracing for impact) can reduce the magnitude of the head's kinematic response in a population of 46 contact sport athletes (male and female) aged between 8 and 30 years [40]. Another study on a group of 49 football players (high school/collegiate) has shown that the odds of sustaining higher magnitude head impacts are reduced with better cervical strength and lower angular displacement following impact [42]. However, their findings did not show that stronger and larger neck muscles in players decreased head impact severity [42]. Meanwhile, Collins et al. (2014) concluded, in their study, which included 6704 high school athletes, that neck strength can be a valuable screening tool to prevent concussion [43]. Further research is needed to clarify these hypotheses and the actual role of neck strength in reducing risk of concussion.

A study by Leddy et al. (2012) for mTBI subjects slow to recover shows that a lightweight level of exercise can be beneficial [44]. Kozlowski et al. (2013) recently published a cross-sectional study about the exercise impact on 34 patients with PCS and a control group of 22 patients [3]. Conclusions showed that patients with PCS had a symptom-limited response to exercise, and the treadmill test was a potentially useful tool to monitor the recovery from PCS [3].

Other physiotherapy treatments, such as vestibular rehabilitation, visual training, cardiovascular training, and the treatment of cervical dysfunctions, have shown some promising avenues, but further studies are needed [4, 10, 14, 17, 20].

## 7. Conclusion

In conclusion, mTBI is a complex injury and needs to be taken care of by different medical specialists working together toward the same goal, recovery [4]. The evidence of cervical spine involvement in mTBI is becoming apparent, but there is a lack of sound evidence in the literature [25]. Our findings have implications for further research. The hypothesis of cervical spine involvement in post-mTBI symptoms and in PCS is supported by increasing evidence and largely accepted. Health professionals should consider assessing the cervical spine of patients affected by mTBI. Some original data articles support this theory and show that persistent headache and postconcussion syndrome are often related to musculoskeletal pathology of the cervical spine. For the mTBI management and treatment, few articles in the literature are available, but well-defined studies showed interesting results about manual therapy (cervical and vestibular) and exercises as effective tools for health care practitioners. Further studies are needed to establish an adequate evaluation and determine guidelines for treatments. The evaluation and treatment of the cervical region are a major step to improve mTBI rehabilitation.

## Figures and Tables

**Table 1:** Preliminary search of literature (total results/abstracts (Abs) selection/articles (Art) for review).

Date		January 1, 1990, to February 16, 2014			February 16, 2014, to May 15, 2014			May 15, 2014, to May 19, 2015		
Resources	Keywords	Total	Abs	Art	Total	Abs	Art	Total	Abs	Art
PubMed	Concussion, Neck	180	12	8	10	0	0	21	5	1
PubMed	TBI, Neck, Treatment	56	0	0	0	0	0	7	2	1
PubMed	TBI, Cervical	83	0	0	33	0	0	17	0	0
PubMed	Concussion, Chiropractic	12	4	1	2	0	0	2	0	0
PubMed	Concussion, Physiotherapy	53	3	2	3	0	0	12	2	1
PubMed	Concussion, Physical Therapy	—	—	—	138	4	1	38	6	1
PubMed	Concussion, Athletic Training, Cervical	30	2	2	0	0	0	6	1	0
ICL	Concussion	32	2	2	0	0	0	4	1	1
SportDiscus	Concussion, Neck	144	0	0	7	0	0	10	4	1
SportDiscus	TBI, Neck, Treatment	5	0	0	0	0	0	0	0	0
SportDiscus	Concussion, Neck, Treatment	15	1	1	4	1	0	3	1	1
SportDiscus	Concussion, Cervical	65	1	1	3	0	0	3	2	1
SportDiscus	Concussion, Chiropractic	9	2	0	0	0	0	2	0	0
SportDiscus	Concussion, Physiotherapy	34	2	2	0	0	0	7	2	1
SportDiscus	Concussion, Athletic Training, Cervical	13	1	1	0	0	0	1	1	1
CINAHL	Concussion, Manipulation	3	3	2	0	0	0	2	1	1
CINAHL	Concussion, Guideline	19	0	0	1	0	0	7	0	0
Cochrane	Concussion	4	1	1	0	0	0	1	1	0
PEDro	Concussion, Physical Therapy	2285	11	5	0	0	0	0	0	0
PEDro	mTBI, Physical Therapy	215	1	1	0	0	0	0	0	0
PEDro	mTBI, Manual Therapy	99	0	0	0	0	0	0	0	0
PEDro	Concussion, Manual Therapy	1132	2	2	0	0	0	0	0	0
PEDro	Concussion, Chiropractic	22	0	0	0	0	0	0	0	0

Total		4510	48	31	201	5	1	143	29	11

**Table 2:** Included studies description.

Authors/sections	Study objective	Population	Methods	Main outcomes/findings
Becker [34]	This review was developed as part of a debate and takes the “pro” stance that abnormalities of structures in the neck can be a significant source of headache	Adult	Literature review	(i) Clinical treatment trials involving patients with proven painful disorders of upper cervical zygapophysial joints have shown significant headache relief with treatment directed at cervical pain generators (ii) Headaches related to cervical spine disorders (cervicogenic headache and chronic headache attributed to whiplash injury) remain one of the most controversial areas of headache medicine (iii) Diagnostic criteria for cervicogenic headache have been developed by the CHISG

Bogduk [30]	To summarize the evidence that implicates the cervical zygapophysial joints as the leading source of chronic neck pain after whiplash	Adult	Narrative review Data were retrieved from studies that addressed the postmortem features and biomechanics of injury to the cervical zygapophysial joints and from clinical studies	(i) Clinical studies have shown that zygapophysial joint pain is very common among patients with chronic neck pain after whiplash (ii) The fact that multiple lines of evidence, using independent techniques, consistently implicate the cervical zygapophysial joints as a site of injury and source of pain strongly implicates injury to these joints as a common basis for chronic neck pain after whiplash

Bonk et al. [38]	To evaluate the effectiveness of conservative management for acute Whiplash-Associated Disorder	Adult	Systematic review and meta-analysis of randomized controlled trials	Improvement of cervical movement in the horizontal plane short term could be promoted by the use of a conservative intervention. The use of a behavioral intervention (e.g., act-as-usual, education, and self-care including regular exercise) may be an effective treatment in reducing pain and improving cervical mobility in patients with acute WADII in the short-medium term

Borich et al. [2]	In this special interest article, we discuss the definition and risk factors associated with concussion, summarize and highlight some of the most widely used assessment tools, and critique the evidence for current principles of concussion management	Adult	Literature review	(i) Disease Control and Prevention describes *mild traumatic brain injury *(mTBI which includes concussion) as a silent epidemic (ii) An estimated 1.6 million to 3.8 million sport- and recreation-related brain injuries occur in the United States annually, and up to 75% are classified as mild (iii) “Rest” in the form of delaying return to competitive sports may be better served by a universal period of 7 to 10 days than by symptom monitoring, primarily to prevent the potential for reinjury

Brolinson [7]	To systematically review the evidence for rest, treatment, and rehabilitation after sport-related concussion	Sports Adult Pediatric	Systematic review	(i) From 749 articles evaluating rest and 1,175 evaluating treatment, 2 studies met criteria for the effect of rest and 10 abstracts met criteria for treatment. Three further treatment articles were identified by the authors (ii) Health professionals are more involved in sports and in the concussions follow-up (iii) Interventions included manual spinal therapy, physiotherapy, and neuromotor and sensorimotor retraining compared with rest and graduated exercise, for up to 8 weeks

Collins et al. [43]	To develop and validate a cost-effective tool to measure neck strength in a high school setting and to determine if anthropometric measurements captured by ATs can predict concussion risk	6,704 high school athletes in boys' and girls' soccer, basketball, and lacrosse	Feasibility study Pilot study	(i) Differences in overall neck strength may be useful in developing a screening tool to determine which high school athletes are at higher risk of concussion. Once identified, these athletes could be targeted for concussion prevention programs

Eckner et al. [40]	The purpose of this study was to determine the influence of neck strength and muscle activation status on resultant head kinematics after impulsive loading	46 contact sport athletes 24 males; 22 females aged 8 to 30 years	Descriptive laboratory study	(i) Neck strength and impact anticipation are 2 potentially modifiable risk factors for concussion (ii) The results of this study suggest that greater neck strength attenuates the head's dynamic response to external forces

Fernández De Las Peñas et al. [24]	The aims of the present paper are to detail a manual approach developed by our research group, to help in future studies of the management of the sequels to whiplash injury, and to suggest explanations for the mechanisms of this protocol	Adult	Literature review	(i) The clinical syndrome of whiplash injury includes neck pain, upper thoracic pain, cervicogenic headache, tightness, dizziness, restriction of cervical range of motion, tinnitus, and blurred vision (ii) Spinal manipulation/mobilization and soft tissue mobilization techniques are manual therapies commonly used in the management of neck disorders

Kennedy [14]	This document is intended to provide the user with instruction and direction in the rehabilitation of PCS	Ontario Hospital Canada Adult	PCS and treatment guidelines (i) Cervicogenic (ii) Autonomic (iii) Vestibular (iv) Vision (v) Education	(i) Anatomically, the cervical spine is closely linked to structures that can cause many of the same symptoms as concussion (ii) PCS treatment has traditionally consisted of rest, education, neurocognitive rehabilitation, and antidepressants with limited effectiveness (iii) Balance deficits and postural instability are commonly reported after concussion

Gravel et al. [36]	This systematic review investigated the effectiveness of interventions initiated in acute settings for patients who experience mTBI	Adult	Systematic review Cochrane's risk of bias assessment tool	(i) According to the published literature, no intervention initiated acutely has been clearly associated with a positive outcome for patients who sustain mTBI, and there is little evidence suggesting that follow-up interventions may be associated with a better outcome (ii) There is a paucity of well-designed clinical studies for patients who sustain mTBI. The large variability in outcomes measured in studies limits comparison between them

Guskiewicz et al. [9]	To review the current literature to identify the most sensitive and reliable concussion assessment components for inclusion in the revised version: the SCAT3	Adult	Literature review	(i) One of the major challenges in the medical management of concussion is that there is no single “gold standard” for assessing and diagnosing the injury (ii) Balance deficits or instability are often observable in patients following concussion and the presence of these deficits may be an indicator of vestibular disruption

Hanson et al. [6]	The purpose of this article is to review the current literature in the management and prevention of concussion	Pediatrics	Review	(i) The rise in the number of concussion diagnoses may be due, in part, to increased awareness regarding the potential for complications of concussions and sequelae of multiple concussions, as opposed to an actual increase in the incidence of concussion alone (ii) Typical signs and symptoms of concussion

Harmon et al. [5]	To provide an evidence-based, best practices summary to assist physicians with the evaluation and management of sports concussion* *	Adult Children	Statement of the American Medical Society for Sport Medicine Review	(i) However, as many as 50% of the concussions may go unreported (ii) Headache is the most commonly reported symptom with dizziness the second most common

Headache Classification Committee of the International Headache Society (IHS) [33]	The International Classification of Headache Disorder may be reproduced freely for scientific, educational, or clinical uses by institutions, societies, or individuals	Adult Children	Review guideline	(i) Cervicogenic headache from (1) migraine and (2) tension-type headache includes side-locked pain, provocation of typical headache by digital pressure on neck muscles and by head movement, and posterior-to-anterior radiation of pain (ii) Diagnostic criteria

Hecht [16]	This article reviews the literature on management of posttraumatic headaches, presents an approach to the assessment and treatment of individuals with headaches following TBI that appear to be cervicogenic, focuses specifically on identifying occipital neuralgia, and discusses the technique of occipital nerve blocks	7 males (18–42 yo) 3 females (22–64 yo)	Retrospective review & Report of ten patients	(i) While there are a variety of different posttraumatic headaches, clinicians must be aware of all potential presentations including those emanating from the cervical spine and its affiliated structures (e.g., cervicogenic) (ii) Injury to these structures (innervated by afferent fibres of the 3 sup. cervical roots) (iii) These include but are not limited to muscles, ligaments, vessels, somatic and sympathetic nerves, esophagus, temporomandibular joint, discs, zygapophyseal joints, cervical vertebrae, and the atlantoaxial complex (iv) Whiplash syndrome may be the primary factor in many postconcussive headaches

Hynes and Dickey [12]	To examine the relationship between the occurrence of Whiplash-Associated Disorders and concussion symptoms in hockey players	High school, college/university, Ontario Hockey League, and men's recreational teams (15–35 yo) 20 teams	Prospective study	(i) 183 players were registered for this study; 13 received either a mechanical whiplash injury or a concussion injury (ii) There is a strong association between whiplash-induced neck injuries and the symptoms of concussion in hockey injuries (iii) Acceleration and deceleration of the head and neck complex occurs in sports and can potentially create injuries similar to those incurred in low velocity motor vehicle accidents, as stated in a recent literature review focused on Whiplash-Associated Disorders

King et al. [35]	The objective was to determine the sensitivity, specificity, and likelihood ratio of manual examination for the diagnosis of cervical zygapophyseal joint pain	173 patients with neck pain in whom cervical zygapophyseal joint pain was suspected	Retrospective study	(i) Manual examination had a high sensitivity for cervical zygapophyseal joint pain, at the segmental levels commonly symptomatic, but its specificity was poor (ii) The present study found manual examination of the cervical spine to lack validity for the diagnosis of cervical zygapophyseal joint pain

Kozlowski et al. [3]	To assess exercise intolerance in male and female patients with PCS	34 patients (PCS) 17 males, 17 females Age = 25.9 ± 10.9 22 uninjured individuals	Cross-sectional study* *	(i) Symptoms from concussion typically resolve within 7 to 10 days (ii) The definition of PCS given by the World Health Organization includes a history of traumatic brain injury and 3 or more symptoms (iii) No cognitive testing, exclusion of other disorders, or symptom threshold exists for the diagnosis of PCS (iv) Patients with PCS had a symptom-limited response to exercise, and the treadmill test was a potentially useful tool to monitor the recovery from PCS

Kristjansson and Treleaven [29]	The purpose is to review dizziness in neck pain: implications for assessment and management	Adult	Review	(i) Disturbances to the afferent input from the cervical region in those with neck pain may be a possible cause of symptoms such as dizziness, unsteadiness, and visual disturbances, as well as signs of altered postural stability, cervical proprioception, and head and eye movement control

Leddy et al. [27]	The objective was to compare symptoms in patients with physiologic postconcussion disorder (PCD) versus cervicogenic/vestibular PCD	128 adults	Retrospective review Questionnaire	(i) Clinicians should consider specific testing of exercise tolerance and perform a physical examination of the cervical spine and the vestibular/ocular systems to determine the etiology of postconcussion symptoms (ii) Concomitant injury to the cervical spine resembling whiplash may occur as a result of the acceleration, deceleration forces sustained in concussive trauma (iii) Structural and functional injury to the cervical spine can be associated with prolonged symptoms such as headache, dizziness, blurred vision, and vertigo

Leddy et al. [44]	This review focuses on rehabilitation of concussion and postconcussion syndrome	Adult Children	Review	(i) Early education, cognitive behavioral therapy, and aerobic exercise therapy have shown efficacy in certain patients but have limitations of study design

Leslie and Craton [25]	Based on the current medical evidence, we would suggest that the constellation of symptoms presently defined as concussion does not have to involve the brain	Adult	Editorial comment	(i) Concussion symptoms can emanate from the cervical spine (ii) Whiplash mechanisms of injury are identical to the “impulsive forces” described in concussive injuries (iii) Notably, symptoms such as headache, neck pain, disturbance of concentration or memory, dizziness, irritability, sleep disturbance, and fatigue have been described in both concussion and whiplash patients (iv) Cervical zygapophysial joints have been implicated as generators of headache and dizziness (v) The overlap with neck/whiplash injuries is evident

Lucas [19]	This article reviews the literature on headache management in concussion and mTBI	Adult Pediatric United States	Literature review	(i) Reports of headache after concussion or mTBI in children ranged from 72% to 93% (ii) Headache is one of the most common symptoms after TBI and PTH may be part of a constellation of symptoms that is seen in the postconcussive syndrome

Makdissi et al. [8]	The objectives of the current paper are to review the literature regarding difficult concussion and to provide recommendations for an approach to the investigation and management of patients with persistent symptoms	Adult Sport	Qualitative review	(i) Cases of concussion in sport where clinical recovery falls outside the expected window (i.e., 10 days) should be managed in a multidisciplinary manner by health care providers with experience in sports-related concussion

Marshall [11]	This paper is a review of recent literature on the topic of concussion, consisting of biomechanics, pathophysiology, diagnosis, and sideline management	Athletes United States	Narrative review	(i) The cervical spine not only is a potential source of injury that we must be aware of but also is implicated as a factor in the concussion itself (ii) Signs and symptoms of concussion from the Association of Sport College of Medicine (ACSM) updated consensus statement

Maugans et al. [22]	The goal of this investigation was to explore cerebral blood flow fluctuation after pediatric sport-related concussion	Twelve children Ages 11 to 15 years Control group	Clinical study	(i) Statistically significant alterations in cerebral blood flow were documented in the sport-related concussion group, with reduction in cerebral blood flow predominating. Improvement toward control values occurred in only 27% of the participants at 14 days and 64% at >30 days after sport-related concussion

McCrory et al. [4]	The new 2012 Zurich Consensus statement is designed to build on the principles outlined in the previous documents and to develop further conceptual understanding of this problem using a formal consensus-based approach	International consensus (i) Adults (ii) Pediatric	International consensus Sport concussion	(i) An initial period of rest may be of benefit (ii) Multimodal physiotherapy treatment for individuals with clinical evidence of cervical spine and/or vestibular dysfunction may be of benefit (iii) Persistent symptoms (>10 days) are generally reported in 10–15% of concussions. In general, symptoms are not specific to concussion and it is important to consider other pathologies (iv) PCS should be managed in a multidisciplinary manner by health care providers with experience in sports concussion

Mihalik et al. [39]	The objective was to evaluate the effect of cervical muscle strength on head impact biomechanics	37 volunteer ice hockey players Age = 15.0 ± 1.0 years	Prospective cohort study	(i) The hypothesis that players with greater static neck strength would experience lower resultant head accelerations was not supported (ii) There is still nonempirical support for the role neck musculature may play in reducing the risk of mild TBI that is worthy of investigation in a young at-risk sample

Moser et al. [21]	The objective of this article is to evaluate the efficacy of cognitive and physical rest for the treatment of concussion	High school and collegiate athletes (*N* = 49) Range = 14–23 yo Mean = 15.0 yo 67% male 33% female	Retrospective analysis	(i) Participants showed significantly improved performance on Immediate Post-Concussion Assessment and Cognitive Testing and decreased symptom reporting following prescribed cognitive and physical rest (ii) These preliminary data suggest that a period of cognitive and physical rest may be a useful means of treating concussion-related symptoms, whether applied soon after a concussion or weeks to months later

Pelletier [18]	The purpose of this paper is to present a review of the diagnosis and treatment of the potentially catastrophic neck and head injuries caused by spearing in Canadian amateur football	Amateur football United States and Canada	Literature review	(i) Associated cervical trauma with concussion may include one or several of neck pain, reduced cervical range of movement, cervicogenic headache, cervicogenic vertigo, and occipital neuralgia (ii) Several manual techniques for the treatment of posttraumatic concussion syndrome have been described as either “direct” or “indirect”

Reid et al. [37]	This study aimed to determine the efficacy of sustained natural apophyseal glides (SNAGs) in the treatment of this condition	34 adults 17 SNAGs 17 Placebo	Double-blind randomized controlled clinical trial	(i) The SNAG treatment had an immediate clinically and statistically significant sustained effect in reducing dizziness, cervical pain, and disability caused by cervical dysfunction

Schmidt et al. [42]	The purpose of this study was to compare the odds of sustaining higher magnitude in-season head impacts between athletes with higher and lower preseason performance on cervical muscle characteristics	49 high school and collegiate American football players	Cohort study	(i) The study findings showed that greater cervical stiffness and less angular displacement after perturbation reduced the odds of sustaining higher magnitude head impacts; however, the findings did not show that players with stronger and larger neck muscles mitigate head impact severity (ii) Male athletes also exhibit greater stiffness and capacity to store elastic energy compared with female athletes

Schneider et al. [26]	The objective of this study was to determine the risk of concussion in youth male hockey players with preseason reports of neck pain, headaches, and/or dizziness	3832 males Ice hockey players (11–14 yo) 280 teams	Prospective study	(i) Preseason reports of neck pain and headache were risk factors for concussion (ii) Dizziness was a risk factor for concussion in the Pee Wee nonbody checking (iii) A combination of any 2 symptoms was a risk factor in the Pee Wee nonbody checking cohort and the Bantam cohort (iv) Neck pain is the third most commonly reported baseline symptom in varsity athletes

Schneider et al. [10]	The objective of this study was to determine if a combination of vestibular rehabilitation and cervical spine physiotherapy decreased the time until medical clearance in individuals with prolonged postconcussion symptoms	18 males 13 females 12–30 years	Randomized controlled trial	(i) A combination of cervical and vestibular physiotherapy decreased time to medical clearance to return to sport in youth and young adults with persistent symptoms of dizziness, neck pain, and/or headaches following a sport-related concussion (ii) The cervical spine is cited as a source of pain in individuals with whiplash (iii) The upper cervical spine can cause cervicogenic headaches (iv) A combination of manual therapy and exercise has been shown to be more effective than passive treatment modalities in individuals with neck pain

Scorza et al. [13]	Current concepts in concussion	Children adolescents	Literature review	(i) Initial evaluation involves eliminating cervical spine injury and serious traumatic brain injury (ii) Selected symptoms of concussion

Signoretti et al. [15]	The following review represents the authors' effort to piece together the current concepts and the most recent findings about the complex basic physiology underlying the injury processes of this particular type of brain trauma and to emphasize the nuances involved in conducting research in this area	European countries United States Adult	Literature review	(i) Postconcussive symptoms may be prolonged in a small percentage of cases, but the acute clinical symptoms largely reflect a functional disturbance rather than a structural injury, which usually is confirmed by the absence of abnormalities on standard neuroimaging studies (ii) The symptoms of concussion reflected a functional disturbance rather than a structural damage such as contusion, hemorrhage, or laceration of the brain

Smith et al. [32]	This preliminary study examined a sample of individuals who did and did not respond to facet block as well as healthy controls to determine whether there were differences in their physical and psychological features once the effects of the blocks had abated and symptoms had returned	58 adults (18–65 yo) Calgary, Canada	Cross-sectional study	(i) Following FB procedures, both WAD groups demonstrated generalized hypersensitivity to all sensory tests, decreased neck ROM, and increased superficial muscle activity with the CCFT compared to controls (ii) Both WAD groups demonstrated psychological distress (iii) Chronic WAD responders and nonresponders to feedback (FB) procedures demonstrate a similar presentation of sensory disturbance, motor dysfunction, and psychological distress. Higher levels of pain catastrophization and greater medication intake were the only factors found to differentiate these groups

Spitzer et al. [28]	The purpose was to expose the clinical classification of Whiplash-Associated Disorders	Adult	Guideline	(i) Grades 0 to 4 (clinical presentation)

Stovner et al. [23]	A main objective of this study was to assess the validity of this diagnosis by studying the headache pattern of concussed patients that participated in one historic (*n* = 131) and one prospective cohort (*n* = 217) study	200 patients (18–67 yo) Trauma involving LOC of < 15 min. Kaunas, Lithuania	Questionnaires study (after 3 months & 1 year)	(i) Existence of pretraumatic headache was a predictor of posttraumatic headache, although pretraumatic headache seems to have been underreported among the concussed patients (ii) This is negative correlation, and the lack of specificity indicates that headache occurring 3 months or more after concussion is not caused by the head or brain injury (iii) Rather it may represent an episode of one of the primary headaches, possibly induced by the stress of the situation

Tator et al. [1]	This report is intended to improve understanding of the epidemiology of neurological conditions and the economic impact on the Canadian health care system and society	Adult Children Canada	Statistical report	(i) Cerebral concussions are commonly known as mild traumatic brain injury (mTBI) (ii) By contrast, Statistics Canada estimated in recent studies that the annual incidence for mTBI is 600 per 100,000 persons and 11.4 per 100,000 inhabitants for a traumatic brain injury (TBI) (iii) However, the highest age group incidence is between 19 and 29 years, representing approximately one-quarter of patients with cranial trauma (iv) Children (18 years and younger) represented almost 45% of the patients with head injury

Tierney et al. [41]	The purpose was to determine whether gender differences existed in head-neck segment kinematic and neuromuscular control variables responses to an external force application with and without neck muscle preactivation	20 females 20 males Adult	Cohort study	(i) Gender differences existed in head-neck segment dynamic stabilization during head angular acceleration (ii) Females exhibited significantly greater head-neck segment peak angular acceleration and displacement than males despite initiating muscle activity earlier (SCM only) and using a greater percentage of their maximum head-neck segment muscle activity

Treleaven et al. [31]	This study measured aspects of cervical musculoskeletal function in a group of patients (12) with postconcussional headache (PCH) and in a normal control group	8 males (15–48 yo) 4 females (20–44 yo)	Retrospective study	(i) Twelve of the 15 eligible patients consented to enter the study (ii) The most frequent major symptomatic segments were C1-C2, C2-C3, C0-C1, and C3-C4. Signs of cervical articular and muscular dysfunction distinguished the PCH group from the control group (iii) As upper cervical joint dysfunction is a feature of cervicogenic causes of headache, the results of this study support the inclusion of a precise physical examination of the cervical region in differential diagnosis of patients suffering persistent headache following concussion

Watanabe et al. [20]	The specific goals of this review include (1) determination of effective interventions for PTH (2) development of treatment recommendations (3) identification of gaps in the current medical literature regarding PTHA treatment (4) suggestions for future directions in research to improve outcome for persons with PTHA	Adult Child	Literature review The level of evidence: American Academy of Neurology criteria	(i) Head pain may be related to direct damage to the skull or brain tissue; muscular, tendinous, and/or ligamentous injury to the cervical spine; and injuries to peripheral nerves. Other nervous system injuries, such as visual and vestibular system damage, also may contribute to headache syndromes (ii) Biologically based interventions included a variety of biofeedback mechanisms, physical therapy and manual therapy, immobilization devices, ice, and injections

Weightman et al. [17]	The purpose of this article is to provide a summary of the development process and to share specific recommendations for PT practice with service members who sustain MTBI	Military and civilian populations	Literature review MTBI-related, evidence-based reviews and guidelines	(i) Determine the disability and its severity related to the neck, jaw, and headaches (ii) Physical therapy interventions with the strongest evidence in the treatment of PTH include a multimodal approach of specific training in exercise and postural retraining, stretching and ergonomic education, and manipulation and/or mobilization in combination with exercise

**Table 3:** Most common symptoms of mTBI according to their categories [4, 6, 11–13].

References	Cognitive	Somatic physical	Affective emotional	Sleep disturbance
Marshall, 2012 [11] Hanson et al., 2014 [6] Hynes and Dickey, 2006 [12] Scorza et al., 2012 [13] McCrory et al., 2013 [4]	(i) Confusion (ii) Retrograde amnesia (iii) Anterograde amnesia (iv) Loss of consciousness (v) Disorientation (vi) Feeling foggy (in a fog) (vii) Vacant stare (viii) Inability to focus (ix) Delayed verbal responses (x) Delayed motor responses (xi) Slurred (xii) Incoherent speech (slurred) (xiii) Excessive drowsiness	(i) Headache (ii) Dizziness (iii) Balance disruption (iv) Nausea (v) Vomiting (vi) Visual disturbances (vii) Phonophobia	(i) Emotional liability (ii) Irritability (iii) Fatigue (iv) Anxiety (v) Sadness	(i) Trouble falling asleep (ii) Decreased sleep (iii) Increased sleep

Hanson et al., 2014 [6]	(i) Feeling slowed down (ii) Difficulty concentrating (iii) Difficulty remembering (iv) Forgetting of recent information (v) Confused about recent events (vi) Answers questions slowly (vii) Repeats questions	(i) Balance problems (ii) Visual problems (iii) Fatigue (iv) Sensitivity to light (v) Sensitivity to noise (vi) Dazed (vii) Stunned	(i) Nervousness	(i) Drowsiness

Hynes and Dickey, 2006 [12]	(i) Dysphagia (ii) Seeing stars	(i) Deafness (ii) Ringing in the ears (iii) Temporomandibular		

Scorza et al., 2012 [13]	(i) Disorientation (ii) Stunned (iii) Vacant stare	(i) Blurred vision (ii) Convulsions (iii) Light-headedness (iv) Numbness (v) Tingling (vi) Tinnitus	(i) Clinginess (ii) Depression (iii) Personality changes	

**Table 4:** Summary table of the Whiplash-Associated Disorder (WAD) classifications and concussion symptoms that can manifest themselves in any grade of WAD [6, 20, 27, 28].

WAD classification	Symptoms
0	No neck complaints
I	Complaint of neck pain, stiffness, or tenderness
II	Neck complaint with musculoskeletal signs
III	Neck complaint, musculoskeletal signs, and neurological signs
IV	Fracture and/or dislocation

## Appendix

See Figure 1 and Tables 1, 2, 3, and 4, and also see “Summary of the Cervicogenic Headache (CGH) Diagnostic Criteria [33]” in Section 6.2.

## Abbreviations

mTBI: Mild traumatic brain injury
ICL: Index to Chiropractic Literature
PEDro: SportDiscus, Physiotherapy Evidence Database
CINAHL: Cumulative Index to Nursing and Allied Health Literature
CDC: Centers for Disease Control and Prevention
TBI: Traumatic brain injury
PCS: Postconcussion syndrome
MRI: Magnetic Resonance Imaging
WAD: Whiplash-Associated Disorder
IHS: International Headache Society
PTH: Posttraumatic headache
CGH: Cervicogenic headache
GCS: Glasgow Coma Score
SAC: Standardized Assessment of Concussion
BESS: Balance Error Scoring System.
